# Trend and Monitoring of Skin Autofluorescence in Patients Receiving Hemodialysis

**DOI:** 10.1016/j.ekir.2024.06.016

**Published:** 2024-06-12

**Authors:** Natasha J. McIntyre

**Affiliations:** 1 London Health Sciences Centre and Lawson Health Research Institute, London, Ontario, Canada

See Clinical Research on Page 2110

All cause and cardiovascular mortality is significantly increased in people receiving maintenance hemodialysis (HD), compared to the general population.^1^ A contributing factor to the development of vascular damage is the formation and accumulation of advanced glycation end products (AGEs) formed by the nonenzymatic reaction of reducing sugars with free amino groups of proteins, lipids, and nucleic acids. Their formation is increased due to oxidative stress. Hyperglycemia and the interaction of blood coming into contact with an HD dialyzer membrane, have also been identified as contributing to an increase in AGE formation in people on dialysis. Subsequent accumulation is further aggravated by the fact that AGEs are normally excreted via the kidney. Patients with end-stage kidney disease receiving HD are therefore subject to particularly high levels of tissue bound AGEs because of their increased formation and reduced clearance.^2^

A simple noninvasive method of measuring AGE deposition within the tissues, has been developed over recent years using skin autofluorescence (SAF). There is a growing body of evidence to attest that SAF, as a marker of AGE accumulation, is a risk factor for all-cause mortality, cardiovascular mortality, and kidney function decline in multiple types of kidney disease.^3^ Previous cross-sectional studies have shown that a high baseline SAF has a strong association with increased mortality in people on dialysis^2^; however, there has been a paucity of evidence regarding the impact of changes over time in SAF and mortality risk in patients on HD. One study reported an increase in SAF predicting a higher mortality in patients on HD^4^; however, this finding was based on interval SAF measurement readings only, at baseline and 1 year. These findings run the risk of being subject to regression to the mean (i.e., results may be influenced by an element of chance) and limiting confidence in the results.

In a recent article, Viramontes Horner *et al.**^5^* have provided observations derived from an observational study on 120 patients undergoing HD. SAF measurements were measured at 6 time points over 24 months (baseline, 3, 6, 9, 12, and 24 months), calculating the SAF trend in order to derive a robust rate of change over time. This study builds on this group’s long standing research interest in this area and represents the first study to collect SAF data at multiple time points, thus addressing the challenge posed by regression to the mean. In addition to being unique in its assessment of SAF over time, it also identifies both higher baseline SAF and SAF trend as independent risk factors for increased all-cause mortality in the population receiving HD. SAF trend was associated with a 2.4 times higher risk of overall mortality after 5 years of follow-up. Interestingly, the authors did not find any significant associations between SAF trend and other, more traditional risk factors, such as smoking, diabetes, dialysis vintage, cardiovascular disease, C-reactive protein, and serum creatinine; suggesting that, in patients receiving HD, SAF provides additional information over and above identifying additional conditions associated with cumulative metabolic stress.

As highlighted earlier, multiple mechanisms may explain the association between increased SAF and mortality. It has been noted that this association may be, in part, due to an association between SAF and malnutrition, with malnutrition being well-documented to be common and a significant factor for poor survival on dialysis. This association may be because of the complex overlapping mechanisms contributing to both observations; namely, oxidative stress rapidly forms AGEs and promotes systemic inflammation and *vice versa*. Oxidative stress and chronic inflammation, in combination with other mechanisms can lead to loss of appetite, hypoalbuminemia, and ultimately malnutrition.^6^ Malnutrition may also contribute to the formation of AGEs due to interaction with inflammatory and oxidative processes. Thus, the association between SAF, malnutrition, and increased mortality may be explained by the creation of a vicious cycle between oxidative stress, chronic inflammation, and malnutrition. In a previous study, this group has also shown both SAF and malnutrition to be independent predictors of higher mortality in patients on dialysis, and not SAF just being a surrogate marker of malnutrition.^7^ In light of this growing body of literature, the collection of SAF in patients receiving maintenance HD may be useful in both risk prediction, but also in monitoring response to interventions designed to target the malnutrition inflammatory complex.

The limitations in the use of SAF are well-described in the paper. In particular, the well-documented issue about the reliability of SAF measurement in people with dark skin types (Fitzpatrick skin types V–VI), effectively eliminating people with these skin types from consideration. This is a significant barrier that the authors and many others agree needs to be overcome to enable the equitable implementation of any monitoring strategies, with attempts to innovate and improve noninvasive SAF measurement to be encouraged. SAF is validated on the volar (palm side) forearm, with the measurement being performed at the same location allowing comparison. There has been a suggestion that less pigmented areas, such as palms, may not be suitable due to varying thickness of the epidermal layer in different people. However, 1 study found that using a novel SAF instrument, SAF was significantly higher at palmoplantar sites in Asian subjects with type 2 diabetes, as compared to those without.^8^ At present though, the challenging interpretation of SAF values in those with very dark skin, continues to be unaddressed. There is some suggestion that the use of polarizers might enhance the measurement of autofluorescence from the dermis by eliminating surface reflection when crossed, thus enhancing the SAF measurement, especially in very dark-skinned subjects.^9^ However, further studies focusing on these specific issues are required, to be able to support the inclusion of people with very dark skin types in monitoring practices.

Given the complexity of the interactions of many factors driving poor outcomes in patients receiving HD, SAF as a cumulative measure of multiple drivers, is an attractive and intriguing area for future study to try and further improve personalized patient care delivery.

## Disclosure

The author declared no competing interests.
